# PBX1: a TALE of two seasons—key roles during development and in cancer

**DOI:** 10.3389/fcell.2024.1372873

**Published:** 2024-02-09

**Authors:** Laura Crisafulli, Matteo Brindisi, Mirko Giuseppe Liturri, Cristina Sobacchi, Francesca Ficara

**Affiliations:** ^1^ IRCCS Humanitas Research Hospital, Milan, Italy; ^2^ Milan Unit, Istituto di Ricerca Genetica e Biomedica (IRGB), National Research Council, Milan, Italy

**Keywords:** PBX1, TALE, development, cancer, t(1;19), Myeloproliferative Neoplasm (MPN), Hematopoietic stem cells (HSC)

## Abstract

Pre-B cell leukemia factor 1 (PBX1) is a Three Aminoacid Loop Extension (TALE) homeodomain-containing transcription factor playing crucial roles in organ pattering during embryogenesis, through the formation of nuclear complexes with other TALE class and/or homeobox proteins to regulate target genes. Its contribution to the development of several organs has been elucidated mainly through the study of murine knockout models. A crucial role for human development has been recently highlighted through the discovery of different *de novo* pathogenic PBX1 variants in children affected by developmental defects. In the adult, PBX1 is expressed in selected tissues such as in the brain, in the gastro-intestinal and urinary systems, or in hematopoietic stem and progenitor cells, while in other organs is barely detectable. When involved in the t(1;19) chromosomal translocation it acts as an oncogene, since the resulting fusion protein drives pre-B cell leukemia, due to the induction of target genes not normally targeted by the native protein. Its aberrant expression has been associated to tumor development, progression, or therapy-resistance as in breast cancer, ovarian cancer or myeloproliferative neoplasm (MPN). On the other hand, in colorectal cancer PBX1 functions as a tumor suppressor, highlighting its context-dependent role. We here discuss differences and analogies of PBX1 roles during embryonic development and in cancer, focusing mainly on the most recent discoveries.

## Introduction

PBX1 belongs to a class of homeobox transcription factors called TALE, due to a Three Aminoacid Loop Extension domain located in the homeodomain. The class is composed of two families, the MEINOX (which includes MEIS and PREP proteins) and the PBC (which includes PBX1-4). All TALE transcription factors contain an atypical homeodomain, which allows binding to DNA and to other transcription factors, and two protein-protein interacting domains, which mediate binding to a large variety of HOX proteins and/or to mutually exclusive MEINOX proteins. Like other TALE members, PBX1 is also able to recruit chromatin accessibility regulators, such as the SWI/SNF complexes, and co-repressors, such as histone deacetylases (Longobardi et al., 2014). Therefore, depending on the context, PBX1 can either promote or repress transcription of target genes. According to the current view, context-specific roles are provided by interaction with selected cofactors that confer tissue or cell specificity, by the cis-regulatory landscape, and by the co-expression of other transcription factors that are part of the same gene regulatory network. In addition to acting in concert with different HOX and other TALE-homeodomain partners, PBX1 is also found in “HOX-less” domains (Capellini et al., 2011; Losa et al., 2018). Examples of non-HOX partners are MYOD, whose interaction with the PBX1-MEIS1 complex drives transcription of the Myogenin gene during skeletal muscle differentiation (Berkes et al., 2004), or SMAD4, which, in complex with PBX1 and PREP1, mediates Activin-dependent expression of the FSHβ gene (Bailey et al., 2004), important for ovulation and spermatogenesis. Moreover, in accordance with its expression in embryonic stem cells (ESC) (Chan et al., 2009), PBX1 has been proposed as a “pioneer factor” (Sagerström, 2004), although unequivocal demonstration is still missing; it marks selected genes for transcriptional activation through specific histone binding in closed chromatin, to increase DNA access for other transcription factors and determining cell fate changes, such as in the context of specification of the skeletal muscle and neuronal lineages (Grebbin and Schulte, 2017).

PBX1 has a widespread distribution in many tissues and in different steps of development (Selleri et al., 2019). In human adults it is mainly expressed in glandular tissues, female tissues, the bladder and in subsets of hematopoietic cells (Nagel et al., 2021; Mary et al., 2022). Different isoforms are produced due to alternative splicing, although the function of some of them is unknown. PBX1a is mainly expressed in the brain and in adulthood, PBX1b is typical of the embryo, while PBX1d is expressed in CD4^+^ T cells. Some PBX1 functions are shared with other PBX family members, mainly PBX2, while others are peculiar of PBX1 (Capellini et al., 2006; Selleri et al., 2019).

PBX1 has long been known as a developmental regulator; its role has been dissected mainly through constitutive and conditional mouse models, as well as through compound mutants of PBX1 and one of the other PBC family members or one of his molecular partners. However, there is increasing evidence that its functions during murine development are conserved in humans (Slavotinek et al., 2017). On the other hand, PBX1 aberrant expression is linked to cancer in several of the tissues in which it plays a role during development, as discussed below.

## PBX1 as a developmental regulator

Several studies of *Pbx*-mutant mouse embryos have revealed PBX1 as a crucial developmental regulator. Among the other PBC family members, only PBX1 is absolutely required for embryonic development since other PBX knockout embryos are viable. PBX1 role has been linked to hematopoietic development (DiMartino et al., 2001), neuronal and cardiovascular patterning (Chang et al., 2008; Vitobello et al., 2011; Sgadò et al., 2012), lung (Li et al., 2014) and diaphragm formation (Russell et al., 2012), pancreas development (Kim et al., 2002), spleen ontogeny (Brendolan et al., 2005), urogenital differentiation (Schnabel et al., 2003a; Schnabel et al., 2003b), face morphogenesis (Losa et al., 2018), skeletal and limb patterning (Selleri et al., 2001; Capellini et al., 2011), among others. More recently, its role in limb morphogenesis including the cell type (mesoderm progenitors) and the time window (initiation of hindlimb bud development) that requires PBX function has been further elucidated through a multi-omics approach (Losa et al., 2023). PBX1 also controls self-renewal and pluripotency of human ESCs by directly regulating NANOG expression (Chan et al., 2009); it is downregulated during ESC early differentiation, but expressed again in ESC–derived hematopoietic stem/progenitor cells (Oshima et al., 2011).

Several of the functions of PBX1 during development that have been discovered through murine models have some correspondence in human developmental anomalies caused by *de novo* PBX1 heterozygous mutations or aberrant expression. A comprehensive overview of all known human developmental defects due to *PBX1* mutations is excellently presented in Mary et al. (Mary et al., 2022). So far, at least forty patients with *PBX1* heterozygous mutations or deletions have been described. This number is likely set to rise thanks to the increasing use of whole-exome sequencing techniques. For example, missense variations cause lung hypoplasia, cardiac malformations, and sexual developmental defects; truncating variants are at the basis of deafness or cryptorchidism. Most frequently, *PBX1* germline variations are associated with kidney syndromic anomalies, often called CAKUTHED for Congenital Anomalies of the Kidney and Urinary Tract (the most frequent birth defects) with or without Hearing loss, abnormal Ears, or Developmental delay; most commonly renal hypoplasia, sometimes with ocular manifestations (Safgren et al., 2022). Other described developmental defects include face, head, and skeletal anomalies. Mutations can be found in one of the protein-binding domains or in the homeodomain, affecting the ability to form heterodimers with protein partners or to bind DNA, respectively. Other mutations affect the nuclear localization or the nuclear export signals, causing cytoplasmic retention (Mary et al., 2022).

PBX1 exerts its role as a developmental regulator also when expressed in non-embryonic tissues. At the maternal-fetal interface, a subset of decidual natural killer (dNK) cells expresses PBX1 at high level (Zhou et al., 2020). In these cells, PBX1 directly regulates the transcriptional expression of growth-promoting factors including Pleiotrophin and Osteoglycin, to allow proper murine fetal growth. Reduced PBX1 activity in human dNK is frequent in women with a history of unexplained recurrent spontaneous abortion, suggesting that the function of PBX1 in dNK cells is conserved in human.

## PBX1 as a regulator of tissue homeostasis

PBX1 acts to establish the proper timing of gene expression also during differentiation of specific cell types beyond morphogenesis and fetal development. For example, in the adult subventricular zone (SVZ) it acts as early regulator of neurogenic cell fate decision and of survival of newly generated neurons, and as pioneer factor for SVZ neurogenesis (Grebbin et al., 2016). In the bone, PBX1, expressed at high levels in osteoprogenitors also in the adult, has been proposed as an attenuator of osteoblast genes transcription through recruitment of chromatin remodeling proteins to the promoters of the osteoblast-related genes osteocalcin and bone sialoprotein. This allows correct gene expression timing and results in matrix maturation and mineral deposition only in fully differentiated osteoblasts, in which PBX1 is no longer expressed, and not in precursors (Gordon et al., 2011), thus contributing to maintaining bone homeostasis. In the hair follicle, PBX1 promotes proliferation, facilitates DNA damage repair, and attenuates senescence and apoptosis of mesenchymal stromal cells (MSCs) (Liu et al., 2019). The homeostasis of the immune system also relies on PBX1 since, together with PREP1, PBX1 mediates the transcriptional activation of IL10 in phagocytes stimulated by apoptotic cells, thus favoring suppression of autoimmunity (Chung et al., 2007). On the other hand, the dominant negative splicing isoform PBX1d, which lacks the DNA and Hox-binding domains, has been recently described as an autoimmunity (Lupus) susceptibility gene that impairs the balance between regulatory and follicular helper CD4^+^ T cells (Li et al., 2020). This isoform is less stable (Park et al., 2023) and has different DNA binding and co-factor recruitment ability relative to the normal isoform; one of its downstream effectors is CD44, a marker of CD4^+^ T cell activation (Niu et al., 2017).

A further example of regulation of tissue homeostasis by PBX1 is its function in early hematopoiesis, as detailed below.

## PBX1 in the hematopoietic system

The role of PBX1 within the hematopoietic system is time- and context-dependent and only seemingly controversial. During embryonic and fetal development it promotes proliferation of stem and progenitor cells and is therefore required for both primitive (Pillay et al., 2010) and definitive hematopoiesis (DiMartino et al., 2001). However, PBX1 role in the developing hematopoietic system is not limited to promoting proliferation in the embryo, but also to specify the Megakaryocyte (Mk) lineage fate and red blood cell development, in complex with its partner MEIS1, through induction of the GATA1 master regulator of erythropoiesis, at the expenses of myeloid induction (Pillay et al., 2010). PBX1 promotes Mk induction also *in vitro* from induced pluripotent stem cells (Cullmann et al., 2021) and from human CD34^+^ cells through direct regulation of PF4 (Okada et al., 2003). PBX1/MEIS1-mediated GATA1 induction has been recently shown to be indirect and to occur through promoting HIF1α transcription (Chung et al., 2021), which is known to regulate erythropoiesis (Zhang et al., 2012). Moreover, PBX1 is essential for lymphoid development starting from the common lymphoid progenitor stage, as demonstrated by elegant RAG1-deficient blastocyst complementation assays (Sanyal et al., 2007). This role is maintained in the adult, impacting on B, T and NK cell number (Xu et al., 2020) and it starts at the level of lymphoid priming in Hematopoietic stem cells (HSCs) (Ficara et al., 2008), although is not apparently linked to proliferation. Similarly, the control of the myeloid vs. erythroid/Mk skewing is maintained in the adult, both in the mouse (Ficara et al., 2013; Muggeo et al., 2021), in which PBX1 is expressed at higher levels in a subgroup of HSCs with Mk potential (Wilson et al., 2015), and in the human (Wang et al., 2022), whereas proliferation promotion does not appear to occur in the adult hematopoietic system at least in steady state. The transition to the adult hematopoietic system is characterized by HSCs becoming quiescent for most of the time, so that their pool is protected by genotoxic stimuli. In this context, rather than acting on proliferation, PBX1 role is linked to preserve HSC potential towards lymphoid, erythroid, and platelet fate at the expenses of other myeloid cell types. Indeed, its absence results in premature myeloid differentiation (Ficara et al., 2013), which might be mediated by downregulating miR-127 (Crisafulli et al., 2019). Interestingly, aberrant expression of PBX1 contributes to the development of cancer involving those lineages (see below).

Overall, PBX1 promotes cell renewal. In some context or cell types, this translates into promoting proliferation; in other contexts, such as in HSCs, preservation of self-renewal capacity is achieved by limiting proliferation.

## PBX1 as cancer contributor

PBX1 functions in the adult are less studied compared to its roles during development. However, its aberrant expression is linked to many different types of cancer.

**FIGURE 1:**
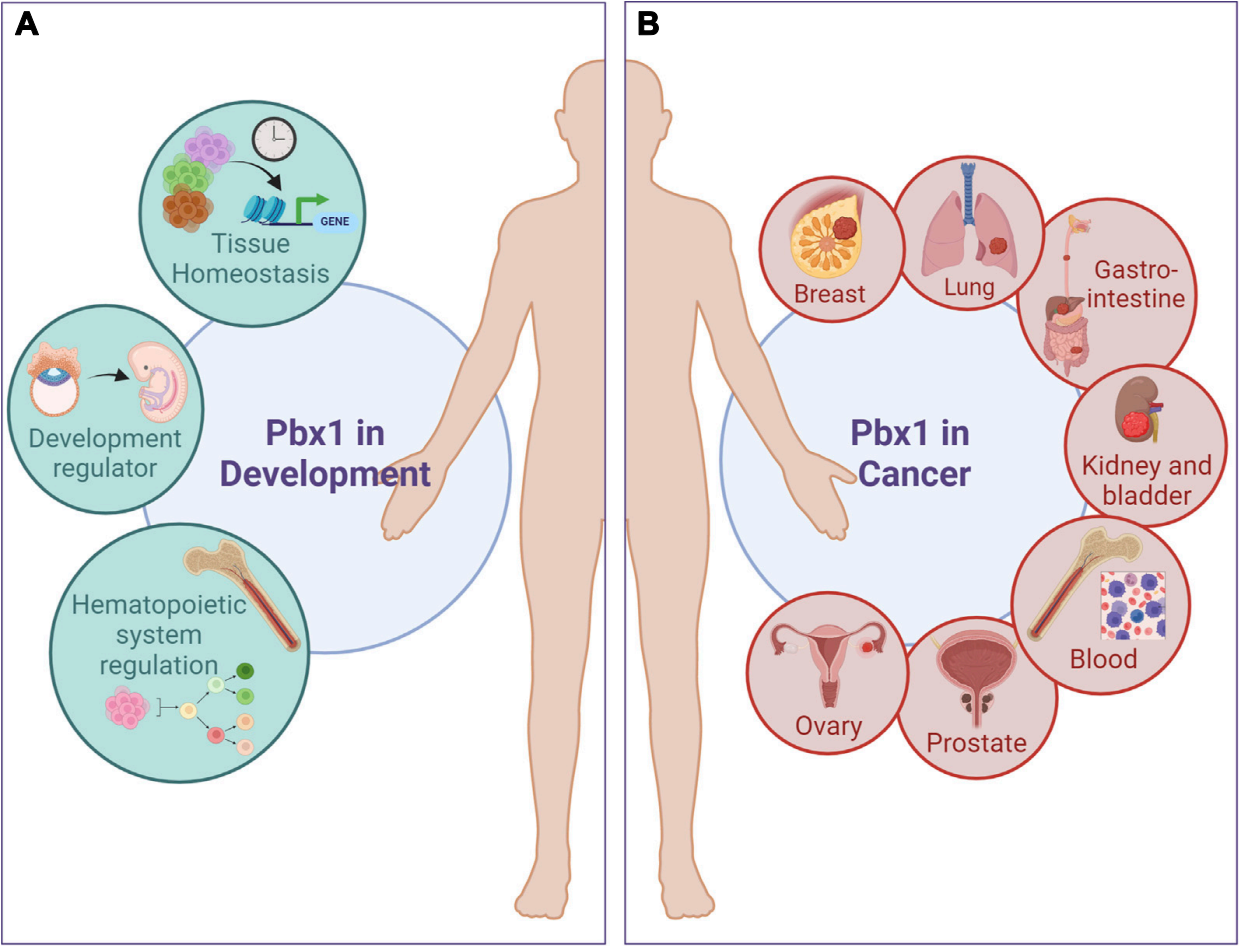
Pbx1 functions in development and cancer. PBX1 regulates the development of several type of cancers involving tissues and cell lineages that are normally regulated during development by PBX1 itself. **(A)**, Green circles represent PBX1 physiological functions; **(B)**, red circles depict some of the tumors in which PBX1 promotes carcinogenesis. A more comprehensive list of PBX1-dependent tumors is present in Table 1. Figure was created with biorender.com.

PBX1 contributes to the development of cancer involving the tissues and cell lineages that it normally regulates during development (Table 1; Figure 1). Examples of affected lineages within the hematopoietic system are B cells, HSCs and Mk-Erythrocyte Progenitors (MEP). The t (Russell et al., 2012; Longobardi et al., 2014) translocation that occurs in pro- or pre-B cells generates the E2A-PBX1 chimeric transcription factor (also named TCF3::PBX1) that contains the N-terminal transactivation domain of the lymphoid lineage E2A transcription factor fused to the C-terminal DNA-binding homeodomain of PBX1. This initiating event, followed by a secondary mutation, results in pediatric B Acute Lymphoblastic Leukemia (B-ALL), accounting for 5%–10% of pediatric ALL (Yin et al., 2023); adult cases have also been reported (Burmeister et al., 2023). E2A-PBX1-dependent gene activation and leukemic cell growth rely on the interaction of the fusion protein with the Mediator complex through the MED1 subunit (Lee et al., 2021) and on RUNX1 coactivation through direct binding to the PBX1 homeodomain (Pi et al., 2020). Moreover, E2A-PBX1-mediated oncogenesis occurs through self-oligomerization, so that dimerization with protein partners that normally stabilize and regulate PBX1 import into the nucleus become dispensable (Lin et al., 2019). E2A-PBX1 fusions have been found prenatally, which might explain the highest occurrence of this type of B-ALL in children (Hein et al., 2019). E2A-PBX1 fusion in isolated cases of B-lymphoblastic lymphomas (B-LBL), rare subtypes of non-Hodgkin lymphoma seen primarily in children or young adults, have also been recently reported (Kubota-Tanaka et al., 2019; Okura et al., 2020). Other genomic alterations occurring in B cells are PBX1 duplications, which give rise to Multiple Myeloma (Trasanidis et al., 2022) or to Hodgkin lymphoma (Nagel et al., 2021; Nagel et al., 2023).

**TABLE 1:** Summary of the role of PBX1 during development, mostly discovered through the analysis of mutant mice or embryos, and role in cancer or other diseases affecting the same tissues whose development depend on PBX1. Most diseases present in the table are discussed or quoted in the text. We apologize for the organs, cells, or diseases that we skipped for brevity.

Tissue/Organ	Role in development or tissue homeostasis	Role in cancer or *other diseases*
Hematopoietic system	Primitive hematopoiesis (Chung et al., 2021); erythropoietic cell lineage specification, myelopoiesis inhibition (Pillay et al., 2010); definitive hematopoiesis (DiMartino et al., 2001)	In a murine model of **Myeloproliferative Neoplasm (MPN)**, it drives thrombocytosis and erythrocytosis; aberrant expression in MPN patients’ cells (Shepherd et al., 2018; Muggeo et al., 2021; Guijarro-Hernández and Vizmanos, 2023)
	Murine Mk development (Pillay et al., 2010; Cullmann et al., 2021); human Mk differentiation (Okada et al., 2003)	
	Postnatal: HSC quiescence and lineage priming; erythroid and lymphoid differentiation (Ficara et al., 2008; Ficara et al., 2013)	
	B cell development (Sanyal et al., 2007)	**Hodgkin lymphoma**: reactivation of B cell progenitor- genes (Nagel et al., 2021); ETS1 inhibition, JAK2 activation (Nagel et al., 2023)
		**B-ALL** (Yin et al., 2023), **B-LBL** (Kubota-Tanaka et al., 2019; Okura et al., 2020): E2A-PBX1 translocation
		**Multiple myeloma**: Expression in plasmacells due to chr1q-amplification; promotes cell cycle (Trasanidis et al., 2022)
	Thymus development (Manley et al., 2004)	**Thymic epithelial tumors:** Copy number gain (Petrini et al., 2013)
		*Autoimmunity*: Impaired Treg (Li et al., 2020a)
	NK development (Xu et al., 2020); fetal growth when expressed in decidual NK cells (Zhou et al., 2020)	*Unexplained recurrent spontaneous abortion* (Zhou et al., 2020)
Cardiovascular	Great-artery patterning and cardiac OFT septation (Chang et al., 2008); angiogenesis (Charboneau et al., 2005)	*Cardiac malformations, heart disease* (Mary et al., 2022)
Skeletal system	Skeletal patterning (Selleri et al., 2001); face morphogenesis by EMT regulation (Capellini et al., 2011)	*Face, head and skeletal anomalies* (Mary et al., 2022)
		*Bone loss* in **Breast Cancer Metastasis** by inhibiting osteoblastogenesis (Liu et al., 2023)
		**Myoepithelial tumors of bone**: EWSR1-PBX1 fusion (Suurmeijer et al., 2020)
Urogenital System–Derivatives of the urogenital ridge	Formation of gonads, Müllerian ducts, mesonephros and kidneys (Schnabel et al., 2003a; Schnabel et al., 2003b); ontology of the mouse and human glomerulus, ureteric branching (Schnabel et al., 2003b; Barry et al., 2019); mouse and human adrenocortical development (Schnabel et al., 2003a; Ferraz-de-Souza et al., 2009) and steroidogenesis	*Sexual developmental defects; CAKUTHED* (Mary et al., 2022)
		**Endometrial Carcinoma**: Tumor suppressor (Guo et al., 2023)
		**Ovarian Cancer**: Oncogene (Park et al., 2008), chemoresistance (Jung et al., 2016)
		**Prostate Cancer**: Proliferation (Kikugawa et al., 2006; Liu et al., 2019b)
		**Renal Clear Cell Carcinoma**: Cell proliferation via JAK2/STAT3 signaling (Wei et al., 2018)
		**Bladder Cancer**: Cell growth, invasion, EMT (Zhao et al., 2022)
Gastrointestinal system/digestive tract	Gut aplasia in Pbx1^−/−^ mice (Selleri et al., 2001)	**Gastric Carcinoma**: potential oncogene (upregulation of miR650 and EMT) (Liu et al., 2021); potential tumor suppressor (upregulation of its PBXIP1 inhibitor) (He et al., 2017)
		**Colorectal Cancer**: Metastasis inhibition (Dai et al., 2023)
		Mouse model of **hepatocellular carcinoma**: tumor suppressor (upregulation of its PBXIP1 inhibitor) (Xu et al., 2013)
	Pancreas and pancreatic islands development (Kim et al., 2002)	*Diabetes mellitus* (suggested) (Kim et al., 2002)
Lung	Lungs development (Li et al., 2014)	Non-Small Cell **Lung Cancer**: Proliferation promotion (Lin et al., 2022) or tumor suppression (Sun et al., 2023)
		*Lymphangioleiomyomatosis*: cell survival by inducing expression of antiapoptotic genes (Olatoke et al., 2023)
		*Lung hypoplasia* (Mary et al., 2022)
Brain	Differentiation of olfactory bulb, mesencephalic and midbrain dopaminergic neurons (Sgadò et al., 2012; Remesal et al., 2020); hindbrain segmentation through control of RA synthesis (Vitobello et al., 2011)	**Brain cancer. Neuroblastoma**: higher expression levels in the initial tumor samples compared with responders (Veselska et al., 2019); **Glioma**: tumor suppressor role) (van Vuurden et al., 2014)
		*Parkinson:* reduced levels in dopaminergic neurons (Villaescusa et al., 2016)
Other ectoderm derivatives	Epithelial cells: Corneal morphogenesis (Murphy et al., 2010); modulation of body-site-specific epidermal barrier (seen with Pbx1 epidermal-specific null mice), auditory sensory epithelium (Li et al., 2020b)	**Oral squamous cell carcinoma**: Oncogene (Platais et al., 2018)
		**Breast Cancer**: involved in lipid metabolism (Wang et al., 2017); regulates the ERα transcriptional response to EGF signaling, driving metastatic progression (Magnani et al., 2011; Magnani et al., 2015); reprogramming of the chromatin landscape (Magnani et al., 2013); prognostic marker (Ao et al., 2020)

PBX1 may contribute to other hematopoietic cancers also in the absence of genomic alterations, for example, if overexpressed or if expressed in HSCs or MEPs in the presence of other driver mutations, such as the somatic V617F mutation in the JAK2 gene typical of myeloproliferative neoplasm (MPN). MPN are heterogeneous blood malignancies associated with increased risk of thrombotic events and of leukemic transformation. They are characterized by the aberrant proliferation of one or more myeloid lineages and progressive bone marrow fibrosis (Coltro et al., 2021). In particular, the polycythemia vera and the essential thrombocythemia subtypes are distinguished by an excess of red blood cells or of platelets, respectively, although the mutation occurs at the HSC or at the progenitors’ level, altering their fate. Since PBX1 is one of the key factors regulating the balance between self-renewal and differentiation in postnatal HSCs (Ficara et al., 2008), and given its action in preserving lymphoid, erythroid and Mk potential (Ficara et al., 2013; Wang et al., 2022), it is not surprising that it exerts a role in blood malignancies in which the cells of origin are HSCs or hematopoietic progenitors, as in myeloid neoplasms. By exploiting an MPN mouse model bearing the JAK2^V617F^ mutation, we demonstrated that PBX1 expression in JAK2^V617F^ HSCs is necessary to sustain MPN (Muggeo et al., 2021), in accordance with a previous report indicating that its overexpression in JAK2^V617F^ HSCs contributes to sustaining an MPN phenotype in animal models (Shepherd et al., 2018). *In silico* analysis revealed an inverse correlation between the genes differentially expressed (DE) in human MPN and those DE in *Pbx1*-null HSCs (Muggeo et al., 2021), providing further evidence of the involvement of PBX1 in human MPN. In addition, PBXIP1 (PBX homeobox interacting protein 1), which codes for a protein that inhibits the transcriptional activation potential of PBX1 by preventing its binding to DNA, is downregulated in MPN patients (Guijarro-Hernández and Vizmanos, 2023). The positive regulation of STAT3 transcription by PBX1 binding to its promoter that has been reported in other tissues (Jung et al., 2016) might represent the underlying mechanism through which its expression contributes to MPN development/maintenance; STAT3, part of the JAK/STAT pathway that acts as an effector of the mutations causing MPN, is widely expressed within the hematopoietic system and its transcription is downregulated in the absence of PBX1 in purified HSCs (Ficara et al., 2008).

The action of another PBX1-MEIS1 downstream effector, the previously quoted HIF1α, might represent another mechanism through which PBX1 exert its role in MPN cells; indeed, HIF1α has been recently proposed as a new potential therapeutic target in these diseases (Baumeister et al., 2020). HIF-1α is a master transcriptional regulator of the response to decreased oxygen levels; it has been shown to regulate HSC quiescence and erythroid differentiation within their hypoxic niche (Takubo et al., 2010; Zhang et al., 2012). HSCs utilize glycolysis as main source of energy prior to maturation and HIF1α is the key transcriptional regulator of glycolytic metabolism in these cells, thus regulating their energy metabolism and differentiation.

Other PBX1 downstream effectors in MPN are the Mk marker CD61, which is downregulated upon PBX1 deletion in the murine MPN model, and the novel early myeloid differentiation marker Embigin, which is instead upregulated upon PBX1 deletion (Muggeo et al., 2021), although direct regulation has not been demonstrated.

Besides the hematopoietic system, PBX1 is involved in several solid tumors. In some instances, increased expression of PBX1 is linked to proliferation promotion, as in breast cancer. Breast cancer represents the major malignancy in women and in approximately two-thirds of cases the pathology is driven by the estrogen receptor ERα (Tyson et al., 2011). By acting as a pioneer factor, PBX1 is essential for the ERα-mediated transcriptional response, promoting greater tumor proliferation and aggressiveness (Magnani et al., 2011; Magnani et al., 2015). A positive correlation of PBX1 and ERα expression levels in breast cancer has been demonstrated. Moreover, depletion of PBX1 inhibits tumor proliferation in the presence of estrogenic stimuli (Magnani et al., 2011).

In addition to acting on primary tumor cells, PBX1 also plays a role in promoting metastasis. In 20%–30% of cases, ERα-positive breast cancer patients treated with endocrine therapy eventually relapse and progress to metastatic disease (Musgrove and Sutherland, 2009). The acquisition of resistance to endocrine therapy is a long-term sequential process accompanied by an important transcriptional reprogramming of the cell, suggesting that remodeling of the chromatin landscape may play a central role in this process. In this context, PBX1 was shown to promote resistance to endocrine therapy *in vitro* by controlling the expression of several genes implicated in this process (Magnani et al., 2013).

Metastasis, including bone metastasis, is the main cause of disease-related mortality in breast cancer. When breast cancer cells colonize the bone, they release cytokines such as osteopontin and RANKL, which act on osteoclast precursors promoting their maturation into bone resorbing osteoclasts (Granata et al., 2022). In addition, exosomes released by breast cancer cells contain reduced levels of miR-6881-3p, recently shown to target PBX1 in osteoblasts thus negatively regulating the expression of its downstream effectors. This results in reduced osteoblastogenesis that likely further contributes to bone loss, although this mechanism has not been shown *in vivo* yet (Liu et al., 2023). Therefore, PBX1 upregulation is involved in driving tumorigenesis and metastasis in breast cancer by acting on malignant cells as well as in the tumor microenvironment.

The association between PBX1 and estrogen signaling was also confirmed in the estrogen-mediated bladder cancer, in which PBX1 expression levels were positively related to tumor size, lymph node metastasis and poorer survival (Zhao et al., 2022).

Other examples of solid tumors in which PBX1 promotes proliferation include high grade clear renal carcinoma, in which PBX1 was positively related to cell cycle progression and proliferation through the JAK2/STAT3 pathway (Wei et al., 2018). In prostate cancer cell lines, PBX1-HOXC8 heterocomplex formation was shown to drive cell growth (Kikugawa et al., 2006); PBX1-mediated induction of cell proliferation and resistance against anti-cancer drugs was shown also in the patients’ cells (Liu Y. et al., 2019). In ovarian cancer, the association of PBX1 and proliferation was observed to be NOTCH3-dependent (Park et al., 2008), and PBX1-mediated chemoresistance to be related to PBX1 binding to STAT3 promoter, positively regulating its transcription (Jung et al., 2016). Along with these, PBX1 over-expression led to a higher number of *in vitro* colonies in gastric cancer cell lines (He et al., 2017); PBX1 was indeed found to be upregulated in patients’ samples and to promote gastric cancer cell proliferation and invasion through promoting miR-650 transcription (Liu et al., 2021).

Despite PBX1 is a transcription factor, lacking intrinsic enzymatic activity, it has recently been considered as a therapeutic target. One strategy is to destabilize its binding to the DNA or to prevent the formation of hetero-homeodomain PBX1-containing transcriptional complexes, thanks to one or more small molecules. This approach proved to be successful in different cancer cell lines and in an *in vivo* model of ovarian cancer (Shen et al., 2021; Trasanidis et al., 2022). Another strategy is the use of short peptide antagonists able to interfere with PBX-HOX interactions, recently shown to be effective *in vitro* and *in vivo* on lymphangioleiomyomatosis, a rare lung disease that depends on PBX1-HOXD11 interaction (Olatoke et al., 2023). Whether these approaches represent the basis for generating novel drugs to cure human cancer is still to be demonstrated.

## PBX1 as tumor suppressor

In some cancers, decreased expression of PBX1 favors malignancy. This is the case of endometrial carcinoma, in which reduced expression of PBX1 causes increased WNT signaling owing to downregulation of its direct target SFRP4, a known WNT pathway inhibitor (Guo et al., 2023). A similar mechanism occurs in colorectal cancer. In this tumor, lack of PBX1 was shown to cause absence of DCDC2 suppression, resulting in increased WNT signaling and increased spindle function, which in turn lead to cell proliferation and metastasis (Dai et al., 2023). In non-small cell lung cancer PBX1 was recently shown to inhibit tumor growth; in patients’ tissues it is indeed downregulated through binding to the ubiquitin ligase TRIM6, which drives PBX1 proteasomal degradation (Sun et al., 2023). However, this is in contrast with a previous report demonstrating a positive role of PBX1 in cell cycle progression (Lin et al., 2022). Contradictory roles of PBX1 are also present in the framework of gastric cancer. At variance with the previously quoted tumor-inducing role in this type of cancer (He et al., 2017; Liu et al., 2021), other authors reported overexpression of the PBX1 inhibitor PBXIP1 in gastric cancer patients, leading to repressed PBX1 transcriptional activity and promotion of cell proliferation, migration, and invasion (Feng et al., 2015). Other cancers in which PBX1 exert a tumor suppressor role are liver cancer (Xu et al., 2013), glioma (van Vuurden et al., 2014) and oral carcinoma (Platais et al., 2018).

## Conclusion

PBX1 is an important player in development, adult tissue homeostasis and cancer. It can either promote proliferation, as in embryonic/fetal growth and in tumor progression, or act as a brake on cell expansion as in the hematopoietic system or in tumors in which it functions as tumor suppressor. In murine models, its absence results in premature differentiation in different cell types including those belonging to the hematopoietic system (Ficara et al., 2008; Ficara et al., 2013), vascular (Hurtado et al., 2015) and bone tissues (Selleri et al., 2001). Its pleiotropic role, which depends on the cellular context, requires tight regulation. Indeed, both increased or decreased expression leads to disruption of cellular homeostasis and disease, although the mechanisms are not completely clear and deserve further studies.

## Figures and Tables

**Table:** 

**B-ALL**	B Acute Lymphoblastic Leukemia
**B-LBL**	B-lymphoblastic lymphoma
**CAKUTHED**	for Congenital Anomalies of the Kidney and Urinary Tract (the most common birth defects) with or without Hearing loss, abnormal Ears, or Developmental delay
**CD44**	CD44 Molecule
**CD61**	CD61 Antigen. Official symbol: ITGB3 (Integrin Subunit Beta 3)
**E2A**	E2A immunoglobulin enhancer-binding factors E12/E47 (official gene symbol: TCF3)
**ERα**	Estrogen Receptor Alpha. Official symbol: ESR1 (Estrogen Receptor 1)
**ESC**	Embryonic Stem Cells
**FSHβ**	Follicle Stimulating Hormone Subunit Beta
**GATA1**	GATA Binding Protein 1
**HIF1α**	HIF1A, Hypoxia Inducible Factor 1 Subunit Alpha
**HOX**	Homeobox
**HSC**	Hematopoietic Stem Cell
**IL10**	Interleukin 10
**JAK2**	Janus Kinase 2
**MED1**	Mediator Complex Subunit 1
**MEINOX**	contraction of MEIS and pKNOX (Pbx/Knotted homeobox, the PREP gene symbol)
**MEIS**	Meis Homeobox (previously named Myeloid Ecotropic viral Integration Site)
**MEP**	Megakaryocyte-Erythrocyte Progenitors
**Mk**	Megakaryocyte
**MPN**	Myeloproliferative Neoplasm
**MSC**	mesenchymal stromal cells
**MYOD**	Myogenic Differentiation
**NANOG**	Nanog Homeobox
**NK**	Natural Killer
**NOTCH3**	Notch Receptor 3
**PBC**	Regions upstream of the homeodomain conserved in all PBX family members. Acronym of PBX and ceh20 (the *Caenorhabditis elegans* homolog)
**PBX1**	PBX Homeobox 1, previously named Pre-B-Cell Leukemia Transcription Factor 1
**PBXIP1**	PBX homeobox interacting protein 1
**PF4**	Platelet Factor 4
**PREP**	PBX Regulatory Protein. Official gene symbol: PKNOX
**RAG1**	Recombination Activating 1
**RANKL**	receptor activator of nuclear factor kappaB ligand. Official gene symbol: TNFSF11 (TNF Superfamily Member 11)
**RUNX1**	RUNX Family Transcription Factor 1
**SMAD4**	SMAD Family Member 4
**STAT3**	signal transducer and activator of transcription 3
**SVZ**	subventricular zone
**SWI/SNF**	SWItch/Sucrose Non-Fermentable, ATP-dependent chromatin remodeling complexes composed of several proteins, coded by the SWI and SNF genes
**TALE**	Three Aminoacid Loop Extension
**TCF3**	Transcription Factor 3
